# Inflammation and Oxidative Stress in an Obese State and the Protective Effects of Gallic Acid

**DOI:** 10.3390/nu11010023

**Published:** 2018-12-21

**Authors:** Phiwayinkosi V. Dludla, Bongani B. Nkambule, Babalwa Jack, Zibusiso Mkandla, Tinashe Mutize, Sonia Silvestri, Patrick Orlando, Luca Tiano, Johan Louw, Sithandiwe E. Mazibuko-Mbeje

**Affiliations:** 1 Department of Life and Environmental Sciences, Polytechnic University of Marche, 60131 Ancona, Italy; s.silvestri@univpm.it (S.S.); p.orlando@univpm.it (P.O.); l.tiano@staff.univpm.it (L.T.); 2 Biomedical Research and Innovation Platform, South African Medical Research Council, Tygerberg 7505, South Africa; babalwa.jack@mrc.ac.za (B.J.); johan.louw@mrc.ac.za (J.L.); sithandiwe.mazibuko@mrc.ac.za (S.E.M.-M.); 3 School of Laboratory Medicine and Medical Sciences, College of Health Sciences, University of KwaZulu-Natal, Durban 4000, South Africa; nkambuleb@ukzn.ac.za (B.B.N.); 217063126@stu.ukzn.ac.za (Z.M.); 217063119@stu.ukzn.ac.za (T.M.); 4 Department of Biochemistry and Microbiology, University of Zululand, KwaDlangezwa 3886, South Africa; 5 Division of Medical Physiology, Faculty of Health Sciences, Stellenbosch University, Tygerberg 7505, South Africa

**Keywords:** obesity, insulin resistance, inflammation, oxidative stress, gallic acid, therapeutic target

## Abstract

Metabolic complications in an obese state can be aggravated by an abnormal inflammatory response and enhanced production of reactive oxygen species. Pro-inflammatory response is known to be associated with the formation of toxic reactive oxygen species and subsequent generation of oxidative stress. Indeed, adipocytes from obese individuals display an altered adipokine profile, with upregulated expression and secretion of pro-inflammatory cytokines such as tumor necrosis factor alpha (TNF-α) and interleukin (IL-6). Interestingly, natural compounds, including phenolic enriched foods are increasingly explored for their ameliorative effects against various metabolic diseases. Of interest is gallic acid, a trihydroxybenzoic acid that has progressively demonstrated robust anti-obesity capabilities in various experimental models. In addition to reducing excessive lipid storage in obese subjects, gallic acid has been shown to specifically target the adipose tissue to suppress lipogenesis, improve insulin signaling, and concomitantly combat raised pro-inflammatory response and oxidative stress. This review will revise mechanisms involved in the pathophysiological effects of inflammation and oxidative stress in an obese state. To better inform on its therapeutic potential and improvement of human health, available evidence reporting on the anti-obesity properties of gallic acid and its derivatives will be discussed, with emphases on its modulatory effect on molecular mechanisms involved in insulin signaling, inflammation and oxidative stress.

## 1. Introduction

**Figure 1:**
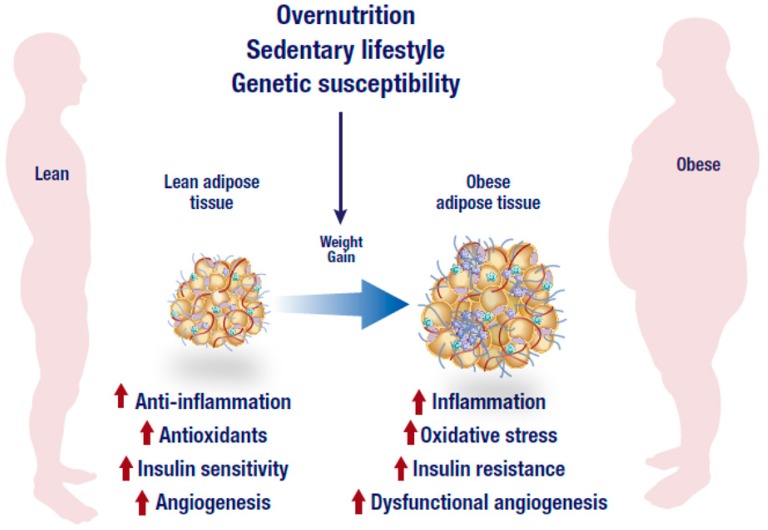
Overnutrition, sedentary lifestyle and genetic susceptibility are the leading factors associated with the development of obesity. In addition to dysfunctional angiogenesis, an obese state is characterized by an abnormal inflammatory response, low antioxidant capacity and reduced insulin sensitivity that may eventually lead to the generation of inflammation, oxidative stress and insulin resistance. The figure was modified from the following website, https://mexicobariatriccenter.com/improve-adipose-tissue-function/.

Global estimates show that more than 1.9 billion adults are overweight, while over 600 million of these individuals are classified as obese [1]. The rising trend in the incidence of overweight and obesity is not only limited to developed countries as factors such as urbanization and unhealthy lifestyle, which contribute to its significant rise are also prominent in developing nations [1]. In fact, sub-Saharan women are far more likely to be obese than men, which further affects pregnancy and maternal health [1]. This can translate to complications and adverse effects on infant health, as previously hypothesized [2]. Visceral obesity is associated with the development of chronic metabolic diseases including insulin resistance, type 2 diabetes (T2D), and cardiovascular disease [3]. The mechanism linking obesity to these comorbidities has not been fully elucidated. However, a growing body of knowledge suggests that a possible convergence of an inflammatory state, which results in chronic inflammation and oxidative stress that is localized within an adipose tissue (Figure 1). Adipose tissue inflammation plays a crucial role in promulgating obesity-related metabolic complications including the development of insulin resistance [4,5]. An imbalance between energy intake and expenditure results in adipose tissue expansion due to excessive lipogenesis in adipose tissues [6].

Adipose tissue is regarded as an endocrine organ that plays a pivotal role in the development of obesity. As excessive fat accumulation in the adipose tissue is associated with weight gain [6]. Over the years, different kinds of adipocytes have been characterized and these include beige, white, and brown which can occur in diverse proportions within individual depots, and their presence has been associated with mixed health outcomes. For example, while excessive storage of white adipose tissue is linked to detrimental effects through its aberrant secretion of pro-inflammatory cytokines, brown adipose tissue is unique for containing abundant mitochondria that are essential for improving cellular respiration and increasing adaptive thermogenesis [7,8]. Adipocytes secrete various endocrine factors such as adiponectin, estrogen, leptin, and an array of cytokines. The type of cytokines released, depend on the systemic or intracellular levels that may modulate various cell signals that can either prevent or exacerbate metabolic complications [8]. Some of the prominent mechanisms that are modulated by various endocrine factors in an obese state include insulin signaling, adipogenesis, pre-adipocyte proliferation and differentiation, and the regulation of mitochondrial energy dissipation through the modulation of lipid metabolism. For this reason, systemic or intracellular control of these factors has been an ideal therapeutic target aimed at preventing obesity or ameliorating its associated complications.

The use of natural products as therapeutic agents in preventing metabolic disease has become popular. Despite the fact that medicinal plants have been used for centuries to combat various ailments [9], it is only in the past few decades that we have seen a rapid rise in studies reporting on the metabolic disease preventative capacity of several plant bioactive compounds or naturally derived products, as reviewed elsewhere [10]. For example, the health benefits of plant phenolics are well established, which may be attributed to their known antioxidant, anti-inflammatory, signal transducing and other biological capabilities [10,11,12,13]. Such plant phenolics include gallic acid, a trihydroxybenzoic acid found in a variety of foods and herbs that are increasingly studied for its biological activities [14,15,16,17]. Certainly, there has been an increase in the experimental data evaluating the ameliorative effects of gallic acid on metabolic diseases, including obesity [17,18,19,20,21,22]. Furthermore, several reviews focusing on the therapeutic potential of gallic acid have also been published. Briefly, in 2013, Locatelli et al. [23] focused on alkyl esters of gallic acid as anticancer agents. In 2015, Badhani et al. [24] gave an overview of the therapeutic and industrial applications of gallic acid, mostly focusing on its antioxidant properties. In the same year, Choubey et al. [25] summarized evidence of patents reporting on anticarcinogenic, antimicrobial, antimutagenic, antiangiogenic and anti-inflammatory properties of gallic acid and its ester derivatives. In 2016, Fernandes and Salgado reviewed analytical methods for the determination and quantification of gallic acid, including emphasizing the advantages and limitations of each technique [26], while Nayeem et al. [27] gave a general overview on the therapeutic potential of gallic acid. In 2017, Kosuru et al. [28] discussed literature summarizing the effects of gallic acid and gallates in human health and disease, with specific emphasizes on mitochondria as the target site. 

Although the aforementioned reviews have provided an important platform that improves our understanding on the therapeutic potential of gallic acid and its derivatives, none have appraised literature on the anti-obesity properties of this phenolic acid. The current review systematically extracted the available primary findings and critically assessed these studies to better inform on the anti-obesity properties of gallic acid by modifying an already published protocol [29]. For data extraction, a search on the association between gallic acid and obesity was conducted using major search engines and databases such as PubMed/Medline, EMBASE, Cochrane Library Databases and Google Scholar. The search was done from inception until end of June 2018, grey literature including abstract proceedings and pre-prints were also included. There were no language restrictions applied, while review articles were assessed for primary findings. Medical subject heading (MeSH) terms such as gallic acid and its derivatives, metabolic syndrome, obesity, inflammation, oxidative stress, and apoptosis, including corresponding synonyms and associated terms for each item were used. Plants and extracts not reported to contain gallic acid, or that through background check had not been characterized to contain gallic acid or its derivatives, were excluded from this study. Furthermore, pathophysiological mechanisms involved in an obese state, especially the detrimental effects of enhanced pro-inflammatory response and oxidative stress are discussed to highlight the anti-obesity potential of gallic acid.

## 2. Inflammation and Insulin Resistance in Adipose Tissue

**Figure 2:**
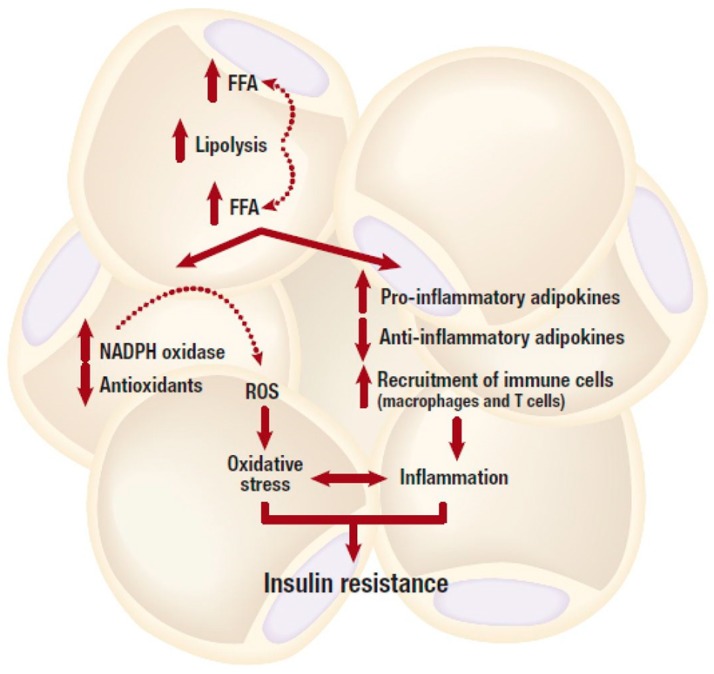
An obese state is associated with dysfunctional lipid metabolism including excessive lipolysis, which in turn leads to increased production and secretion of free fatty acids (FFAs). Elevated FFA levels can cause an abnormal pro-inflammatory response, and subsequent development of insulin resistance. Whereas, depleted intracellular antioxidant systems in the adipose tissue, mainly due to increased production of reactive oxygen species (ROS) can generate oxidative stress, and this can further lead to the development of insulin resistance. NADPH, nicotinamide adenine dinucleotide phosphate.

Generally, it is well accepted that adipose tissue expansion in an obese state is accompanied by elevated inflammation and infiltration of inflammatory macrophages into adipose tissue. As displayed in Figure 1, increased abdominal adipose tissue accelerates the production of pro-inflammatory cytokines which are associated with the degree of metabolic dysfunction [30]. Adipose tissue is highly vascularized and angiogenic [31]. This ensures adequate neovascularisation that is required for oxygen and nutrient supply of the expanding tissue. An imbalance between expansion and vascularization results in hypoxia, which promotes adipose tissue inflammation. Through the reduction of angiogenetic growth components such as vascular endothelial growth factor (VEGF) during hypoxia, several processes including the formation of new blood cells in the adipose tissue are hindered [32,33]. Adipose tissue expansion is usually accompanied by reduced vascularization, and this process may exacerbate metabolic disease pathogenesis [32,33]. In fact, effective modulation of angiogenesis and vasculatures in adipose tissue has been proposed to be a viable mechanism to reverse obesity associated complications [34]. However, uncontrolled adipose tissue expansion in an obese state is also associated with dysfunctional lipid metabolism including excessive lipolysis (Figure 2), which in turn leads to increased production and secretion of free fatty acids (FFAs) into the circulation [35]. Inflammation localized in adipocytes, alters their adipokine profile, which may shift towards a pro-inflammatory phenotype that is accompanied by a high expression and secretion of pro-inflammatory cytokines such as tumor necrosis factor-alpha (TNF-α), interleukin-6 (IL-6) and other mediators of inflammation [36]. TNF-α is one of the earliest pro-inflammatory cytokines identified and its abnormally elevated levels are associated with obesity, insulin resistance and T2D. For example, knockout of TNF-α in diet-induced obese or leptin deficient (*ob*/*ob*) mice was linked with increased insulin sensitivity [37,38]. Such effects have also been confirmed in human subjects and leptin resistant mice where elevated lipids or TNF-α have been associated with obesity, insulin resistance and cardiovascular complications [39,40,41], suggesting that adipose tissue inflammation and obesity are implicated in the development of T2D.

Macrophage infiltration into the adipose tissue can also initiate chronic immune activation, leading to metabolic dysregulation and an increased risk of cardiovascular disease [42,43]. Several factors, either derived from adipocytes or endothelial cells within adipose tissue, are thought to initiate the recruitment of macrophages into adipose tissue. This leads to the infiltration of some immune cells, such as neutrophils and T cells which subsequently induces hypoxia and adipocyte cell death [38]. The order of immune cell recruitment remains unclear however in obesity, macrophages represent more than half of leukocyte population present in visceral and subcutaneous adipose tissue [30]. Some studies have demonstrated a direct association between elevated macrophages found in visceral white adipose tissue and increased body mass index [44]. In animal models of diet induced obesity, macrophages constitute around 50% of all adipose tissue cells [30], whereas in lean mice and humans, adipose tissue cells comprise of only 5% macrophages [30]. In fact, inhibiting macrophage infiltration by blocking the monocyte chemoattractant 1 (MCP-1) ameliorates insulin resistance [45]. 

Adipokines such as adiponectin have been demonstrated to inhibit macrophage function [46,47] and leptin has been shown to promote inflammation by inducing T lymphocyte activation and proliferation [48]. Products of lipolysis such as FFAs activate T lymphocytes which result in increased adipose mass and adipose tissue inflammation. Interestingly, T-helper cell 17 (T_H_17) cytokine levels have been connected with inflammation in obese people living with T2D [49]. On the other side, it has been found that hyperglycemia induces the production of TNF-α through the down-regulation of monocyte cell surface CD33, a transmembrane receptor expressed by monocytes in peripheral blood [50]. CD33 plays a crucial role in inhibiting cytokine production, and the reduction of CD33 expression in monocytes and lymphocytes is associated with increased production of inflammatory cytokines such as TNF-α and IL-1 [50,51]. T_H_17 lymphocytes secrete IL-17, which triggers the nuclear factor kappa-light-chain-enhancer of activated B cells (NF-κB) leading to the activation of B lymphocytes [52].

In relation to insulin signaling (Figure 2), high levels of FFA and pro-inflammatory adipokines have been reported to induce insulin resistance in insulin sensitive cells such as adipocytes, hepatocytes and cardiomyocytes [35,53,54]. This is mediated by inhibiting the insulin signaling pathway through the activation of intracellular stress kinases such as the inhibitor κB kinase (IKK) complex and c-JUN NH_2_-terminal kinase (JNK) [55,56]. Subsequently, this can induce either inflammation or the serine phosphorylation of insulin receptor substrate 1 (IRS-1), leading to impaired downstream insulin signaling [55,56]. Chronic levels of FFAs and pro-inflammatory cytokines can also activate the inducible nitric oxide synthase (iNOS), which prompts nitric oxide (NO) production thereby causing a subsequent degradation of IRS-1 [57]. Furthermore, NO also blocks phosphatidylinositol 3-kinase (PI3K)/protein kinase B (Akt) activity by inducing s-nitrosylation of Akt [58]. The excessive production of saturated FFAs increases the accumulation of toxic lipid metabolites such as ceramides, diacylglycerols, linoleic acid, or phosphatidic acid, and activate phosphokinase C (PKC) [53]. Phosphorylation of this kinase enzyme induces downstream activation of IKK and JNK, and this may lead to a subsequent interruption of the insulin signaling and generation of oxidative stress [53]. This has been demonstrated in experimental models either suppressing or overexpressing JNK [59]. 

## 3. Oxidative Stress in Adipose Tissue

In addition to driving an enhanced pro-inflammatory response, adipose tissue expansion during the progression of obesity can result in excess production of toxic radical species that can cause generation of oxidative stress. Although mechanisms involved in this process are complex, a strong correlation between reduction of the vasculature (vessel rarefaction) and generation of oxidative stress through reactive oxygen species (ROS) has been reviewed [60,61]. Besides their well-known detrimental actions, ROS are physiologically important for acting as second messengers in cell signaling and they also play a pivotal role in cellular homeostasis [62]. The term ROS encompasses free radical species, including hydroxyl (·OH), superoxide (O_2_^• −^), and hydrogen peroxide (H_2_O_2_). Oxidative stress is a consequence of an imbalance between ROS production and scavenging, while chronic or sustained oxidative stress may be associated with cellular damage by oxidizing cellular constituents such as proteins, lipids and DNA [62,63]. In adipose tissue, obesity can induce oxidative stress mainly via catalytic activity of nicotinamide adenine dinucleotide phosphate (NADPH) oxidase (NOX) enzyme or through dysfunctional mitochondrial oxidative phosphorylation [63,64]. NOX remains the major route for ROS production in adipocytes [65]. This plasma membrane-bound enzyme contributes to ROS production by transferring electrons from NADPH to oxygen, thus generating O_2_^• −^, which is further converted to H_2_O_2_ by superoxide dismutase [65]. NOX exists in seven different isoforms that are widely expressed in various tissue. Notably, NOX4 is predominantly expressed in murine and human adipocytes [65]. In obese mice, the mRNA expression levels of NOX subunits were solely increased in adipose tissue and this was accompanied by increased ROS production in adipose tissue [66]. While adipose-specific deletion of NOX4 attenuated adipose tissue inflammation and the early onset of insulin resistance in diet-induced obese mice [67], suggesting that NOX4 derived oxidative stress and ROS production plays a role in the development of insulin resistance in adipose tissue. High levels of FFAs and glucose, which are abundant in obesity seem to contribute to NOX activation and ROS production (Figure 2). In cultured 3T3-L1 adipocytes, high levels of FFAs and glucose increased ROS production via NOX activation [66,68]. In addition, treatment with NOX inhibitors or the silencing of NOX4 appeared to ameliorate this effect by decreasing ROS generation [66,68]. 

The mitochondrial electron transport chain is among the main sites for ROS production in most mammalian cells that mainly takes place during oxidative phosphorylation [69]. Several studies have shown that mitochondrial-derived ROS production is associated with the late stages of obesity as compared to NOX-derived ROS production, which is associated with the early stages of obesity [64]. In a morbidly obese state, adipocytes utilize FFAs derived from triglyceride stores via excessive lipolysis for energy production, as a result of glucose deprivation due to insulin resistance [64]. Excessive FFAs lead to an overflow of electrons in the electron transport chain during oxidative phosphorylation, resulting in their leakage and generation of O_2_^• −^ followed by the production of other ROS molecules [64,70]. Excess production of mitochondrial derived ROS is associated with aggravation of inflammation and development of insulin resistance in adipocytes through the activation of the NF-κB [71]. The phenomenon of enhanced pro-inflammatory response and oxidative stress in an obese state contributes significantly to the development of other metabolic complications such as T2D, cardiovascular diseases and certain types of cancers [3,72]. Hence the increased focus on developing therapeutic agents that target inflammation and oxidative stress with the aim of preventing these diseases [72,73,74,75]. In addition to well-established antidiabetic drugs such as metformin and insulin, literature on the anti-inflammatory and antioxidant effects of some other agents like salsalate, diacerein and chloroquine has been previously reviewed [76]. Briefly, in addition to their beneficial effects in maintaining blood glucose levels in diabetic patients, most of these drugs prompt diverse effects ranging from reducing circulating oxidized low-density lipoprotein-induced pro-inflammatory responses in monocytes and macrophages, to inhibiting IL-1β, TNF-α, and NF-κB levels in blood and different body tissues. However, limitations, such as scanty studies on human subjects, as well as controversial and inconclusive evidence, indicate the need to investigate alternative therapies. Interestingly, increasing research shows that gallic acid can ameliorate inflammation and oxidative stress, through improvement of mitochondrial biogenesis, among discussed mechanisms [28]. 

## 4. A Brief Overview of the Classification, Occurrence, and Bioavailability of Gallic Acid

**Figure 3:**
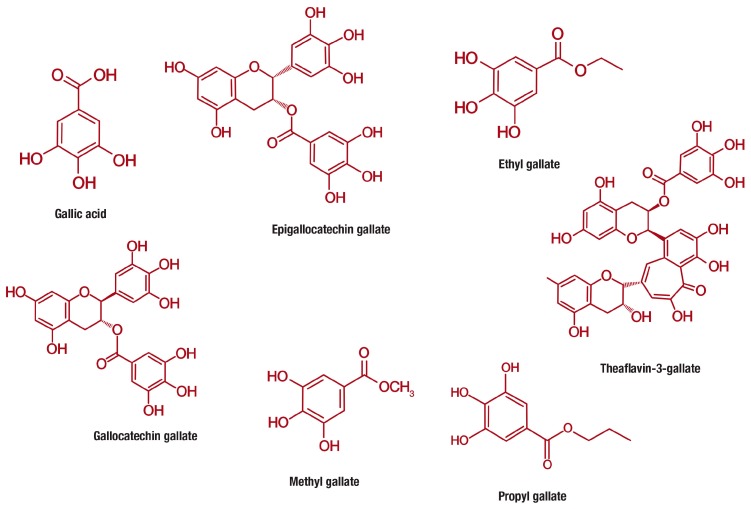
Chemical structures of gallic acid and its derivative compounds, including epigallocatechin gallate, ethyl gallate, gallocatechin gallate, methyl gallate, propyl gallate, theaflavin-3-gallate that are increasingly studied for their anti-obesity properties.

Gallic acid (PubChem CID: 370), is a 3,4,5-trihydroxybenzoic acid with the molecular formula C_7_H_6_O_5_ (MW 170.12 g/mol) that is abundantly found in gallnuts, sumac, witch hazel, tea leaves, oak bark, and other plants [77]. Gallic acid belongs to a distinct group of naturally occurring compounds known as phenolic acids, and is conventionally produced by hydrolysis of tannic acid. This class of compounds is unique for containing a phenol ring that possesses at least one carboxylic acid functionality (Figure 3). Phenolic acids are generally subclassified into benzoic acids comprising seven carbon atoms (C6-C1) and cinnamic acids with nine carbon atoms (C6-C3) [12]. However, gallic acid exist predominantly as hydroxybenzoic acids [77,78] and occurs in different forms of esters and salts, including epigallocatechin gallate (PubChem CID: 65064) [79], ethyl gallate (PubChem CID: 13250) [80], gallocatechin gallate (PubChem CID: 199472) [81], methyl gallate (PubChem CID: 7428) [82], propyl gallate (PubChem CID: 4947) [83], theaflavin-3-gallate (PubChem CID: 169167) [84] and others (Figure 3). 

Despite their wide distribution, the health effects of phenolic acids, including gallic acid, can be affected by several factors including poor stability as well as restricted bioavailability and absorption [85,86]. It is mostly accepted that bioavailability can vary among different phenolic acids, and the dietary abundance of a specific compound does not necessarily translate to best bioavailability profile. Although available experimental studies in animals and humans have demonstrated that gallic acid can be absorbed in the body [85,87,88,89], its effectiveness can be hindered due to rapid metabolism and elimination [85,86]. Furthermore, like most natural products, additional studies specific to determining food species with properties that elevate gallic acid bioavailability, and knowing how much of certain foods one need to consume to have the beneficial dosage of this phenolic acid in plasma are required. Nonetheless, after oral administration, it is estimated that approximately 70% of gallic acid is absorbed and then excreted in the urine as 4-*O*-methylgallic acid [89,90]. Most importantly, several methods have been tested in efforts to improve the bioavailability of gallic acid in the circulation and target tissues. These include repeated dosing and the use of structural analogs or derivative compounds of gallic acid, which significantly improves the plasma levels of this phenolic acid [91]. Similarly, other researchers showed that using other systems such as phospholipid complexation or microencapsulation can enhance the therapeutic efficacy of gallic acid through increasing absorption and bioavailability in serum [92]. Recently, it has been shown that gallic acid significantly enhanced the bioavailability of diltiazem, a calcium channel blocker widely used to treat hypertension, leading to the inhibition of both cytochrome P450 isozyme (CYP3A)-mediated metabolism and P-glycoprotein-mediated efflux in the intestine and/or liver [87]. This result is of interest since the process of absorption, distribution, metabolism, and excretion of different agents can be affected by co-treatment with other drugs, as well as various physiological and pathological changes. A recent study showed that pharmacokinetic process of gallic acid is different between normal and rats subjected to myocardial infarction [93]. Suggesting that additional studies are required to assess the pharmacokinetic profile of herbal preparations or dietary nutrition containing gallic acid in different pathological conditions, as well as its co-treatment with currently used agents. Nonetheless, experimental data reporting on the ameliorative effect of gallic acid against metabolic complications has increased over the years. 

## 5. Experimental Models Investigating the Anti-Obesity Effects of Gallic Acid

Currently, various experimental models are being explored to investigate the anti-obesity properties of pharmacological compounds, including natural products and plant-derived extracts [94]. Pre-clinical models of obesity are presently divided into different categories, the major ones being based on genetic mutations or manipulation, while others focus on intact animals exposed to obesogenic environments such as being maintained on high-fat diets [94]. Indeed, it was evident that the majority of studies presented in Table 1, Table 2, Table 3 and Table 4 investigated the therapeutic effect of gallic acid or extracts rich in this phenol through the use of fat pads from high fat diet (HFD) fed rats and mice. The only reported transgenic model of obesity used were ddY mice [95], a mouse model known to be susceptible to obese characteristics, including cholesterol, hyperglycemia and hypertriglyceridemia in response to obesogenic diet [95]. Besides the use of cultured 3T3-L1 adipocytes [21,96,97], other models that were widely used to test the anti-obesity properties of gallic acid were in vitro experiments that inhibit various enzymes involved in fat breakdown and metabolism [98,99]. Lipase inhibitors are known to bind to lipase enzymes in the intestine, thus blocking hydrolysis of dietary triglycerides into monoglycerides and FFAs [100]. Recently, in silico methods, such as molecular docking, have also become popular. Such systems have been used to assess the inhibitory effect of gallic acid against lipases [101,102]. 

## 6. Evidence on the Anti-Obesity Properties of Gallic Acid

Although gallic acid was shown to be active against complications such as hemoptysis as early as the 1800s [140], studies reporting on its anti-obesity properties started emerging about three decades ago [113]. A search with the terms “gallic acid and metabolic disease” resulted in approximately 246 articles; however, only 60 studies were specific to gallic acid and its ameliorative effects against obesity associated complications. Data reporting on the ameliorative effect of gallic acid or its derivative compounds, as well as tea, fruits and other plants containing this phenolic acid are summarized in Table 1, Table 2, Table 3 and Table 4, while information on the effect of gallic acid in human studies is presented in Table 5. Information presented in each table includes author details, year of publication, experimental model and dose used, as well as the proposed mechanism of action, if any was investigated. 

Through the use of experimental models discussed above, gallic acid has demonstrated an increased potential to ameliorate a number obesity associated complications, as summarized in Table 1. Concise evidence shows that gallic acid presents with and enhanced effect to reduce body weights in obese rodents [95,105,107]. This effect can either be directly via inhibiting formation of lipid droplets in the liver or adipose tissue, as well as directly by reducing serum levels of triglycerides and low-density lipoprotein [105,106]. In cultured adipocytes or HFD fed rats, such properties have been confirmed [17,18,103], with the modulation of glucose and lipid metabolism implicated as the major mechanism proposed to be involved in the therapeutic benefits of gallic acid. Indeed, the modulatory effect of lipids and glucose intermediates could be related to its effects in improving glucose uptake [109,112], increasing energy expenditure [110], and enhancing insulin sensitivity [20,109]. Albeit regulation of PI3K/Akt signaling could explain its therapeutic potential in enhancing insulin sensitivity [20,112], activation of AMP-activated protein kinase (AMPK) by gallic acid might influence substrate metabolism, as reported elsewhere [111]. Nonetheless, several other natural compounds such as celastrol and resveratrol have been shown to control glucose and lipid metabolism and thereby ameliorate obesity associated complications, including inflammation and oxidative stress through mostly modulating mechanisms such PI3K/Akt and AMPK [147,148]. In any case, although limited information is available on its effect on inflammation, studies summarized in this review support strong ameliorative effects of gallic acid against oxidative stress [104,105,108]. From these studies, enhancing intracellular antioxidants such as glutathione and blocking lipid peroxidation products is linked with reduced oxidative stress.

Furthermore, it appears that increasing adiponectin levels and regulating genes involved in adipogenesis and proliferation may be another mechanism by which gallic acid attenuates obesity associated complications [16,21]. For instance, through upregulation of peroxisome proliferator-activated receptor (PPAR)γ expression and activation of NAD-dependent deacetylase sirtuin-1 (SIRT1)/peroxisome proliferator activated receptor gamma coactivator 1 alpha (PGC1α) pathway this phenolic acid can induce browning of the adipose tissue [111]. It can influence adipogenesis by upregulating protein expression of fatty acid synthase (FAS), FAS ligand (FasL), as well as tumor protein 53 (p53) and activated caspase 3/9 [21]. Interestingly, similar to the mechanism attributed to statin drugs, gallic acid can interfere with cholesterol synthesis by blocking the activity of β-Hydroxy β-methylglutaryl-CoA (HMG-CoA) reductase [108]. However, although data on its comparison with a known antidiabetic agent, pioglitazone [20], there is very limited literature that compares the beneficial effects of gallic acid with widely used anti-obesity or antidiabetic drugs.

## 7. Evidence on the Anti-Obesity Effects of Gallic Acid Derived Compounds

Table 2 summarizes some of the well-investigated derivatives of gallic acid for their anti-obesity properties, including 6-deoxytetra-*O*-galloyl-α-d-glucopyranose, tetra-*O*-galloyl-α-d-xylopyranose, 6-chloro-6-deoxy-1,2,3,4-tetra-*O*-galloyl-α-d-glucopyranose, epigallocatechin gallate, epicatechin-3-gallate, N-(4-(tert-Butyl)phenyl)-3,4,5-trihydroxybenzamide (KMU-3), and methyl gallate [14,17,101,114,115,116,117,118,144,146]. Briefly, some evidence summarized in Table 2 demonstrates that the therapeutic effects of gallic acid were less effective when compared to a few pharmacological compounds, including some of its derivatives. For example, tannic acid displayed better effect in attenuating insulin-stimulated lipogenesis through activation of insulin-receptor-associated tyrosine kinase phosphorylation in Wistar rats [113]. *O*-coumaric acid and rutin displayed a better effect on inhibiting glycerol-3-phosphate dehydrogenase activity, and reducing the expression of PPARγ, CCAAT/enhancer-binding proteins (C/EBP) and leptin in cultured 3T3-L1 adipocytes [96]. Epigallocatechin gallate performed better in decreasing insulin stimulated glucose uptake, with the mechanistic involvement of AMPK pathway [97]. Moreover, (z)-3-(3,4,5-trihydroxybenzoyloxy) propane-1,2-diyl dioleate showed a more pronounced effect than gallic acid in reducing the body weight in Wistar rats [22]. KMU-3 outperformed gallic acid in suppressing lipid accumulation in 3T3-L1 adipocytes by downregulating the expressions of C/EBP-α, PPARγ, and FAS [116]. Although did not show superior effect when compared to gallic acid, the other derivative compounds of this phenolic acid such as 6-deoxytetra-*O*-galloyl-α-d-glucopyranose, tetra-*O*-galloyl-α-d-xylopyranose, epigallocatechin-3-gallate, epigallocatechin 3-*O*-(3-*O*-methyl) gallate and methyl gallate have presented and enhanced effect at improving glucose uptake, inhibiting pancreatic lipase activity, and blocking adipogenesis and proliferation, respectively [14,101,114,115,117,118]. The proposed mechanisms associated with the aforementioned beneficial effects include regulation of CCAAT/enhancer-binding proteins (C/EBPR) and PPARγ expression, as well as stimulation of Wnt/β-catenin signaling to mostly block adipogenesis and proliferation.

## 8. Evidence on the Anti-Obesity Properties of Tea and Fruits Containing Gallic Acid

Table 3 summarizes primary studies reporting on the beneficial effects of tea and fruits containing gallic acid or its derivative compounds against obesity associated complications. Besides tea (*Camellia sinensis*), fruits that have been shown to contain high levels of gallic acid or its derivative compounds include avocado, ellagitannin-enriched polyphenolic extract, *Eugenia dysenterica* DC., freeze-dried jaboticaba peel, grape powder, herbal mixture, *Limonium spp*. (Plumbaginaceae), *Mangifera indica* L., mango, mulberry water, *Nelumbo nucifera* leaf, Norton grape pomace, number ten supplement, *Ocimum basilicum*, pineapple, pomegranate peels, *Psidium guajava* L., Pedro Sato, Paluma and Século XXI, Pu-erh tea, *Punica granatum*, *Ribes nigrum L*, *Rubus suavissimus*, and *Terminalia bellirica* [11,15,19,98,99,102,119,120,121,122,123,124,125,126,127,128,129,130,131,132,133,134,135,136,137,138,139,142,143,145].

From data presented in Table 3, tea appears to be the leading gallic acid-rich product that is explored for its anti-obesity properties. This may be due to the fact that tea is among the world’s most consumed beverage and is increasingly targeted for the treatment of lifestyle diseases [149]. Tea exists in various forms, with green tea prepared in an unoxidized form, oolong partially oxidized, Pu-erh teas requiring boiling water for infusion, while black tea undergoing the complete oxidation process [150]. Although present at varying amounts, all teas contain relatively high levels of catechins and gallic acid [150,151]. Previous reports show that green tea can suppress adipogenesis and lipid synthesis by increasing energy expenditure via thermogenesis, fat oxidation and fecal lipid excretion [152]. Consistently, evidence on this review showed that black and Pu-erh teas have great potential in ameliorating obesity associated complications by mainly reducing visceral fat deposition and lowering hepatic triglyceride levels [15,117,121,122,127,145]. 

Lowering plasma total cholesterol, triglyceride concentrations and low-density lipoprotein-cholesterol levels, in addition to reducing activities of FAS and malic enzyme, are proposed to be the mechanisms involved in the beneficial effects of tea against obesity linked complications. Thus, suggesting that additional studies are required to explore molecular mechanisms involved in the beneficial effect of gallic acid-rich teas against obesity associated complications, especially targeting its role in adipogenesis, insulin signaling, inflammation, and oxidative stress processes.

**Figure 4:**
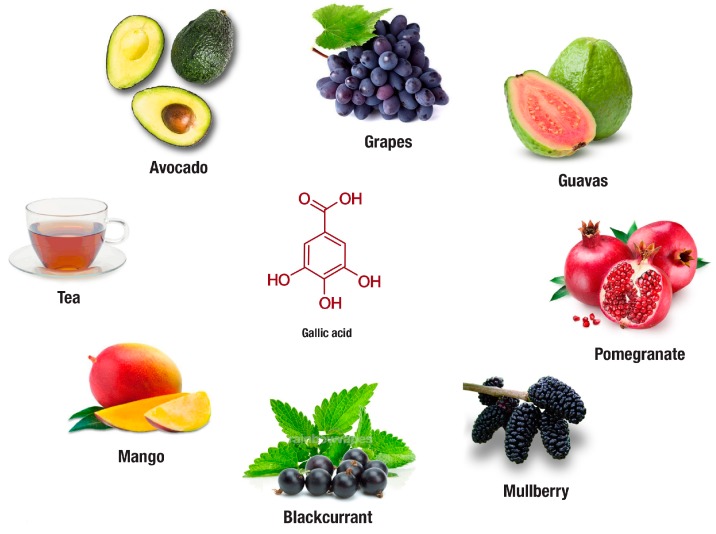
In addition to tea, avocado, blackcurrant, grapes, guava, mango, mulberry and pomegranate are some plants rich in gallic acid or its derivative compounds that are increasingly investigated for their anti-obesity properties. The following websites were used for the extraction of images: Tea, https://www.coffeebean.com/cafe-menu/tea; Avocado, https://draxe.com/avocado-benefits/; Grapes, https://www.indiamart.com/proddetail/purple-grapes-16445565830.html; Guava, https://exoticflora.in/products/guava-red-flesh-fruit-plants-tree; Mango, http://www.adagio.com/flavors/mango.html; Blackcurrant, https://tmbnotes.co/BlackcurrantMentholConcentrate; Mulberry, https://www.amazon.com/Dwarf-Everbearing-Mulberry-Plant-Morus/dp/B008BB8VOW; Pomegranate, https://www.organicfacts.net/health-benefits/fruit/health-benefits-of-pomegranate.html.

In addition to tea, evidence on the therapeutic potential of fruits rich in gallic acid or its derivatives in preventing obesity has also emerged. Fruits of interest include avocado, blackcurrant, grapes, guava, mango, mulberry, and pomegranate (Figure 4). Most of these fruits are commercially available, and their regular consumption has been linked with various health benefits. For instance, avocado (*Persea americana*) extract at 100 mg/kg body weight was found to significantly reduce body mass index, adiposity index, total fat pad mass, blood cholesterol, triglycerides, and low-density lipoprotein in HFD fed rats [124]. Blackcurrant (*Ribes nigrum*) supplemented in diet for eight weeks reduced body weight gain and improved glucose metabolism in HFD fed mice [11]. Although limitations in decreasing oxidative stress in obese female mice have been observed [120], several beneficial effects for grapes (*Vitis vinifera*) extracts have been identified by other researchers in cultured adipocytes and obese rodents [120,129,130]. The beneficial effects include the capacity of this grape extract to reduce plasma C-reactive protein levels, improve glucose uptake and insulin signaling, which may be related to enhanced expression of perilipin-1, fatty acid binding protein 4, GLUT4, as well as PI3K. Another gallic acid-rich fruit, guava (*Psidium guajava* L.), using in vitro-based assays, demonstrated inhibitory effects on lipase, α-glycosidase, α-amylase and trypsin enzyme activities in the presence of simulated gastric fluid [131]. Whereas in cultured adipocytes, mango (*Mangifera indica*) extract showed inhibitory effect against hydrogen peroxide induced production of ROS [128]. On the other hand, mulberry (*Morus alba* L.) extracts supplemented in diet were shown to reduce body weight of obese mice by suppressing visceral fat, accompanied with hypolipidemic effects through the reduction in serum triacylglycerol, cholesterol, and the low-density lipoprotein/high-density lipoprotein ratio [123]. Similarly, using a network-based pharmacological analysis, mulberry extracts have been proposed to regulate TNF-α, PPARγ, glycogen synthase kinase-3 beta (GSK3B), insulin receptor substrate 1 (IRS1), interleukin 6 (IL-6) and other proteins involved in diabetes and obesity associated complications [132]. Last but not least, pomegranate (*Punica granatum*) extracts, using in vitro screening tools have demonstrated an enhanced effect to suppress α-glucosidase, α-amylase, and lipase activities [134]. Overall results presented in this review support the beneficial effects of fruits-rich in gallic acid on ameliorating obesity associated complications [11,120,123,124,128,129,130,131,132,133,134]. However, most of these studies fall short in confirming in vitro findings on other in vivo models, while also demonstrating limitation in unravelling molecular mechanisms by which these fruits can protect against obesity linked anomalies.

## 9. Anti-Obesity Properties of other Plants Rich in Gallic Acid

Besides wine-making products, other plant extracts and products rich in gallic acid or its derivatives include cagaita (*Eugenia dysenterica*), Ceylon cinnamon (*Cinnamomum verum*), jaboticaba (*Plinia cauliflora*), *Limonium*, *Nelumbo nucifera*, *Ocimum basilicum* and *Terminalia bellirica* (Table 4). Through the use of various experimental models these plant extracts display a broad spectrum of ameliorative effects against obesity associated complications. For example, the use of herbal mixture extract rich in gallic acid at 790 mg/kg body weight for 4 weeks improved lipid profile, defective antioxidant stability, and insulin resistance in HFD fed rats [119]. In a similar model of obesity, the use of cagaita extracts at 7 and 14 mg gallic acid equivalent for 8 weeks protected against dyslipidemia, fasting hyperglycemia, and further attenuated both hepatic gluconeogenesis and inflammation as observed by the expression of TNF-α and transcriptional factor NF-κB [19]. Based on in vitro assays, the bark extracts of *Ceylon Cinnamon* showed increased potential to inhibit HMG-CoA reductase, lipase and cholesterol esterase [139]. On the other hand, supplementation with Jaboticaba peel extract for 6 weeks reduced circulating saturated FFAs, blocked lipid peroxidation in the liver and increased its antioxidant defenses in obese rats [136]. Administration of *Nelumbo nucifera* leaf extract mixed at 0.5% in diet for 6 weeks was able to reduce body weight, body lipid accumulation, and the enzymatic activity of FAS, glutamic oxaloacetic transaminase, and glutamic pyruvic transaminase in obese mice [135]. *Limonium spp*. (Plumbaginaceae), a epigallocatechin-rich extract inhibited the activities of pancreatic triacylglycerol lipase, α-amylase and α-glucosidase [137]. Moreover, *Ocimum basilicum* and *Terminalia bellirica* extracts were shown to present with high potential to inhibit the activity of α-glucosidase, α-amylase, lipase, HMG-CoA reductase and angiotensin 1-converting enzyme [98,126,137,138,139]. Inhibition for some of these enzymes, especially lipase may translate to restricted to food absorption resulting in loss of body weight; however in vivo confirmation of such findings is necessary. Anyway, although there is still some difficulty in achieving reduction in body weights in obese rodent models with gallic acid treatment, the overall findings demonstrate that the presence of gallic acid in some plants may enhance their therapeutic effects in preventing obesity associated complications.

## 10. Human Studies Reporting on the Therapeutic Potential of Gallic Acid against Obesity-Associated Complications

Despite the recorded increase in natural product and natural product derived drugs in clinical trials [153], challenges of conducting clinical research of natural products still persists [154]. Toxicity, adverse effects if used on long-term or at the incorrect dosages, and drug-to drug interactions are some of the acknowledged draw backs identified in clinical evaluation of herbal medicine for the treatment of obesity [155,156,157]. Two of the six clinical studies on the anti-obesity properties of gallic acid included in the current review showed that this phenolic acid or its derivatives did not cause weight loss or affect any of the markers assessed except for reducing food intake in obese subjects assessed [142,141]. However, it is of note that although strong evidence linking consumption of natural supplements with effective management of obesity is insufficient, most natural compounds have been specifically credited for attenuating metabolic complications including systemic inflammation and oxidative stress in overweight and obese individuals [158,159,160]. The other four included clinical studies supported the beneficial effect of gallic acid and its derivatives in ameliorating some obesity associated complications [144,143]. These studies showed that in addition to improving the quality of life score of obese patients undergoing biliopancreatic diversion [146], gallic acid-rich extracts can reduce the mean waist circumference, body mass index, and visceral fat values in pre-obese Japanese human subjects [145], and suppress inflammation and oxidative stress associated markers [144,143]. From clinical results summarized in this review (Table 5), it is clear that future work exploring different doses and larger cohorts is required to fully elucidate the therapeutic potential of gallic acid to combat obesity and associated complications in human subjects. Furthermore, a comparison of its effects with other available treatments, such as lipid lowering drugs and other obesity therapies, is still necessary.

## 11. Concluding Remarks

Obesity and the metabolic syndrome are of significant scientific and clinical interest, due to their contribution in the rapid rise of noncommunicable diseases. Although mechanisms describing the pathophysiology of these complications remain complex, inflammation and oxidative stress are understood to be some of the major causal factors implicated in worsening of obesity associated perturbations. Thus, in addition to reducing raised blood glucose or lipid levels, amelioration of inflammation and oxidative stress may be another basic measure taken to improve cellular function in an obese state. At present, only a few therapies are available to improve the lives of obese patients at high risk of developing the metabolic syndrome. To date, some natural products, including gallic acid have been shown to ameliorate complications associated with the metabolic syndrome. This may be through mechanisms involving the reduction of excessive body fat, or ameliorating inflammation and oxidative stress at a cellular level. Certainly, the pre-clinical data summarized in this review support the beneficial effects of gallic acid or its derivatives in preventing obesity-associated complications. Although demonstrated to partially interfere with allergic disorders by acting on G protein-coupled receptor-35 [161], it is still not clear which receptors are targeted by gallic acid or how it could modulate the discussed metabolic benefits. Other interesting questions raised in this review include identification of gallic acid metabolites that may be involved in cellular functions, and investigating its broad effect in increasing angiogenesis or endothelial cell function and thereby reduce oxidative stress. The major shortfalls highlighted in this review include limited to no studies assessing the ameliorative effects of gallic acid against obesity-associated complications in human subjects to confirm its therapeutic potential. This can be further complemented with experiments exploring its concurrent use with current lipid-lowering therapies to investigate whether it would of therapeutic benefit as an adjunct therapy.

## Figures and Tables

**Table 1:** Overview of studies reporting on the ameliorative effect of gallic acid against obesity-associated complications.

Author, Year	Experimental Model, Dose Used, and Intervention Period	Comparative/Combination Therapy	Experimental Outcome and Proposed Mechanism
**Strobel et al., 2005 [103]**	Adipocytes from epididymal fat pads from male Wistar rats treated with gallic acid at 0.1–100 µM	Catechin, myricetin and quercetin were used at 0.1–100 µM, together with 1 µM insulin for 30 s	All compounds inhibited glucose uptake through interfering with the function of glucose transporter (GLUT) 4
**Hsu et al., 2006 [104]**	3T3-L1 pre-adipocytes treated with gallic acid at 43.3 µM for 24, 48 and 72 h	Chlorogenic acid, *o*-coumaric acid and *m*-coumaric acid were used at 72.3, 48.2, and 49.2 µM, respectively, for 24, 48, and 72 h	All phenolic acids, at varying degree, improved the antioxidant status and inhibited proliferation
**Hsu et al., 2007 [21]**	3T3-L1 pre-adipocytes treated with 0.1–250 µM gallic acid for 24, 48, and 72 h	None	Inhibited proliferation by blocking histone deacetylase activity. Further enhanced protein expression of fatty acid synthase (FAS), FAS ligand (FasL), as well as tumor protein 53 (p53) and activated caspase 3/9
**Hsu and Yen, 2007 [105]**	High fat diet (HFD) fed male Wistar rats received 50 and 100 mg/kg body weight of gallic acid for 10 weeks	None	Reduced body weight, organ weight of the liver and adipose tissue weights. Further improved hepatic glutathione levels
**Jang et al., 2008 [106]**	HFD fed female C57BL/6 Cr Slc mice treated with gallic acid, at 1% of diet for 7 weeks	Linoleic acid and a mixture of gallic acid and linoleic acid were mixed with diet	All compounds showed hypolipidemic effects through reducing body weights and hepatic oil droplets, while improving lipid profiles
**Booth et al., 2010 [107]**	Male and female BR VAF/Plus rats given a combination of rhubarb, astragalus, red sage, ginger, and turmeric, together with gallic acid at 215, 430 and 860 mg/kg body weight for 20 days	None	Significantly reduced body weights
**Punithavathi et al., 2011 [108]**	Streptozotocin-induced diabetic male Wistar rats treated with gallic acid at 10 and 20 mg/kg body weight for 21 days	None	Reduced blood glucose and hepatic lipid peroxidation products, glycoprotein components, lipids, and the activity of β-Hydroxy β-methylglutaryl-CoA (HMG-CoA) reductase.
**Oi et al., 2012 [95]**	HFD fed female ddY mice treated with gallic acid at 15, 45 mg/kg body weight for 12 weeks	Black tea extract was used at 50, 100 mg/kg body weight for 12 weeks	Reduced body weights, as well as inhibited pancreatic lipase activity
**Bak et al., 2013 [109]**	HFD fed male C57BL/6 mice treated with gallic acid at 10 mg/kg body weight for 2 weeks	None	Reduction in adipocyte size was associated with upregulation of peroxisome proliferator-activated receptor gamma (PPAR)γ expression and activation of protein kinase B (Akt) signaling pathway
**Ou et al., 2013 [110]**	Oleic acid-induced proliferation of vascular smooth muscle cells treated with gallic acid at 10–30 µM for 48 h	None	Displayed anti-atherogenic effects, inhibited fatty acid synthase (FAS), blocked endothelial nitric oxide synthase and activated 5’ adenosine monophosphate-activated protein kinase (AMPK)
**Chao et al., 2014 [18]**	HFD fed male C57BL/6 mice treated with gallic acid at 50 and 100 mg/kg body weight for 16 weeks	None	Partially reversed metabolic disturbances, including lipid and glucose metabolism, amino acids metabolism, choline metabolism and gut-microbiota-associated metabolism
**Doan et al., 2014 [111]**	HFD fed male C57BL/6 mice treated with gallic acid at 10 mg/kg of body weight for 9 weeks	None	Induced browning of adipose tissue through activation of AMPK/Nicotinamide adenine dinucleotide (NAD)-dependent deacetylase sirtuin-1 (SIRT1)/peroxisome proliferator activated receptor gamma coactivator 1 alpha (PGC1α) pathway. Also regulated uncoupling protein 1
**Gandhi et al., 2014 [20]**	HFD fed and streptozotocin induced diabetic male Wistar rats treated with gallic acid at 20 mg/kg body weight for 28 days	Pioglitazone was used at 10 mg/kg body weight for 28 days	Improved insulin sensitivity through translocation and activation of GLUT4 in phosphatidylinositol -3-kinase (PI3K)/p-Akt dependent pathway. Furthermore, it moderately enhanced PPARγ expression
**Pandey et al., 2014 [17]**	HFD induced male C57BL/6 mice were treated with gallic acid at 2, 4 and 8mg/kg body weight for 28 days	None	Lowered serum levels of triglycerides, and low-density lipoprotein, while increasing high density lipoprotein concentrations
	3T3-L1 adipocytes treated with gallic acid at 3.12, 6.25, 12.5, 25, 50 and 100 µM for 48 h	Aqueous extract *Labisia pumila* and pyrogallol were used at 3.12–100 µM for 48 h	Both compounds and extract showed inhibitory effect on fat droplet formation and triglyceride accumulation
**Makihara et al., 2016 [16]**	3T3-L1 adipocytes were treated with gallic acid at 10–30 µM during differentiation period	Troglitazone was used at 10 μM, while *Terminalia bellirica* hot water extract was used at 0.1, 1.0 and 10 during differentiation	The extract and gallic acid enhanced adipocyte differentiation and adiponectin secretion, partially through increasing adiponectin and fatty acid binding protein-4 levels
**Huang et al., 2018 [112]**	HFD fed male Wistar rats were treated with gallic acid at 10 or 30 mg/kg body weight for 8 weeks	Pioglitazone was used at 30 mg/kg body weight for 8 weeks	Decreased the perirenal adipose tissues and restored expression of insulin receptor and GLUT4 in the perirenal adipose tissues

**Table 2:** Overview of studies reporting on the ameliorative effect of gallic acid derived compounds against obesity-associated complications.

Author, Year	Experimental Model, Dose Used, and Intervention Period	Comparative/Combination Therapy	Experimental Outcome and Proposed Mechanism
**Ong et al., 1995 [113]**	Adipocytes from epididymal fat pads from male Wistar rats treated with gallic acid at 1–1000 µM for various times from 20 min to 2 h	Tannic acid was used at 1–1000 µM for various times from 20 min to 2 h	Tannic acid inhibited insulin stimulated lipogenesis through promoting activation of insulin-receptor-associated tyrosine kinase phosphorylation. Whereas, gallic acid showed no effect
**Ren et al., 2006 [114]**	3T3-L1 pre-adipocytes incubated with 6-deoxytetra-*O*-galloyl-α-d-glucopyranose, tetra-*O*-galloyl-α-d-xylopyranose and 6-chloro-6-deoxy-1,2,3,4-tetra-*O*-galloyl-α-d-glucopyranose at 30 µM for 15 min	None	Improved glucose uptake
**Hsu and Yen, 2007 [96]**	3T3-L1 adipocytes were treated with gallic acid at 1–250 μM for 72 h	*o*-coumaric acid and rutin were used at with 1–250 μM for 72 h	*o*-coumaric acid and rutin demonstrated better effect in inhibiting glycerol-3-phosphate dehydrogenase activity, and the expression of peroxisome proliferator activated receptor (PPAR)γ, CAAT/enhancer-binding proteins (C/EBPR) and leptin. While also upregulating adiponectin levels
**Hsieh et al., 2010 [97]**	3T3-L1 and C3H10T1/2 adipocytes treated with gallic acid at 5–10 μM for 2 h	Compound C, n-acetyl-l-cysteine, epigallocatechin gallate and other catechins, such as epicatechin, epigallocatechin, and epicatechin 3-gallate were used at 5–10 μM for 2 h	Epigallocatechin gallate performed better than other compounds in inhibiting insulin stimulated glucose uptake, with mechanistic involvement of 5’ adenosine monophosphate -activated protein kinase (AMPK) pathways
**Totani et al., 2011 [22]**	High fat diet fed male Wistar rats treated with gallic acid at 90 ppm in diet for 12 weeks	(z)-3-(3,4,5-trihydroxybenzoyloxy) propane-1,2-diyl dioleate (DOGGA) and octyl gallate (OG) were both used at 90 ppm in diet for 12 weeks	DOGGA showed pronounced effect than OG in reducing the body weight in rats. Gallic acid showed no effect
**Sergent et al., 2012 [115]**	In vitro bioassays testing epigallocatechin-3-gallate at 0.8 µM	Kaempferol and quercetin were effective at 13.4 and 21.5 µM, respectively	Epigallocatechin-3-gallate presented pronounced pancreatic lipase inhibitory effect than both kaempferol and quercetin
**Park et al., 2014 [116]**	3T3-L1 adipocytes treated with gallic acid at 30, 60 and 90 µM during differentiation period	KMU-3, a derivative of gallic acid, was used at 1, 5 and 10 µM during differentiation period	KMU-3 outperformed gallic acid in suppressing lipid accumulation in cells. Mechanistically, it inhibited expressions of C/EBP-A, PPARγ, and Fas, as well as some pro-inflammatory markers
**Yang et al., 2015 [117]**	3T3-L1 pre-adipocyte treated with epigallocatechin 3-*O*-(3-*O*-methyl) gallate and epicatechin-3-gallate at 20, 40 and 80 μg/mL for 48 h	None	Epigallocatechin 3-*O*-(3-*O*-methyl) gallate presented higher activity than epicatechin-3-gallate in inhibiting adipogenesis and proliferation
**Jeon et al., 2016 [118]**	3T3-L1 adipocytes treated with methyl gallate at 25, 50 and 75 µM for 48 h	None	Inhibited adipogenesis through stabilizing β-catenin suppression of PPARγ expression. Further stimulated canonical Wnt/β-catenin signaling
**Ediriweera et al., 2017 [14]**	MCF-7 cells treated with gallic acid at 90 µM for 48 h	Ascorbic acid (6.5 µM), catechin (583 µM), curcumin (3.5), epigallocatechin gallate (7.5 µM), and quercetin (70 µM) for 48 h	Only quercetin, curcumin and epigallocatechin gallate showed significant protective effects against leptin-induced proliferation
**Zengin et al., 2017 [101]**	In vitro docking experiments assessing lipase inhibitory effect of gallic acid	*p*-OH-benzoic acid, catechin, epigallocatechin gallate, epicatechin, and rosmarinic acid	Epigallogatechin gallate and rosmarinic acid displayed best docking scores for the inhibition of α-glucosidase, α-glucosidase and lipase activities

**Table 3:** Overview of studies reporting on the ameliorative effect of tea and fruits-rich in gallic acid against obesity-associated complications.

Author, Year	Experimental Model, Dose Used, and Intervention Period	Comparative/Combination Therapy	Experimental Outcome and Proposed Mechanism
**Ikeda et al., 2005 [15]**	High fat diet fed male Sprague Dawley rats treated with tea catechins or heat-treated catechins extracts, which are rich in epigallocatechin gallate and epicatechin gallate at 1% in diet and fed for 23 days	None	Tea and the extracts markedly reduced visceral fat deposition and hepatic triglyceride levels. The activities of fatty acid synthase and malic enzyme were also decreased
**Amin and Nagy, 2009 [119]**	High fat diet fed male albino rats treated with herbal mixture extract rich in gallic acid at 790 mg/kg body weight for 4 weeks	l-carnitine was used at 250 mg/kg body weight for 4 weeks	The extract and carnitine improved disturbed lipid profile, defective antioxidant stability, and high values of insulin resistance parameters
**Hogan et al., 2010 [120]**	High fat diet fed male C57BLK/6J mice treated with Norton grape pomace extract rich in garlic acid at 2.4 g/kg of feed in order to dose each mouse at approximately 250 mg GPE/kg body weight for 12 weeks	None	The extract lowered plasma C-reactive protein levels. However, the extract did not improve oxidative stress as determined by plasma Oxygen Radical Absorbance Capacity (ORAC) assay, glutathione peroxidase, and liver lipid peroxidation
**Cao et al., 2011 [121]**	High fat diet fed male Sprague-Dawley rats treated with Pu-erh tea extract at 0.5 g, 2 g and 4 g/kg body weight for 8 weeks	None	The extract significantly lowered plasma total cholesterol, triglyceride concentrations and low-density lipoprotein-cholesterol levels. It further enhanced mRNA levels of hormone-sensitive lipase
**Chang et al., 2011 [102]**	In vitro molecular docking screening of traditional Chinese medicine, rich in gallic acid, for inhibition of fat mass and obesity-associated protein activity	(S)-tryptophan-betaxanthin, 3-methoxytyramine-betaxanthin, 4-*O*-methylgallic acid, syringic acid, ethacrynic acid, ferulic acid, caffeic acid, canavanine, and 3-methylthymidine	Gallic acid, together with (S)-tryptophan-betaxanthin, 3-methoxytyramine-betaxanthin and 4-*O*-methylgallic acid were among the leading compounds shown to inhibit fat mass and obesity-associated protein activity
**Koh et al., 2011 [122]**	High fat diet fed male Sprague Dawley rats treated with Chinese sweet leaf tea (*Rubus suavissimus*), rich in gallic acid, at 0.22 g/kg body weight for 9 weeks	None	Significantly reduced body weight gain and abdominal fat gain. Although food intake was not affected, blood glucose was lowered, serum triglycerides and cholesterol were significantly reduced
**Peng et al., 2011 [123]**	High fat diet fed male Syrian golden hamsters treated with mulberry water extracts, rich in gallic acid, at 0.5%, 1% and 2% of extract supplemented in diet for 12 weeks	None	The extracts lowered body weight and visceral fat, accompanied with hypolipidemic effects by reducing serum triacylglycerol, cholesterol, free fatty acid, and the low-density lipoprotein/high-density lipoprotein ratio
**Makihara et al., 2012 [98]**	Type 2 diabetic obese male TSOD mice treated with a hot water extract of *Terminalia bellirica*, rich in gallic acid, at 1% and 3% supplemented in diet for 8 weeks	None	The extract displayed preventive effect on obesity, insulin resistance, and hyperlipidemia. It suppressed absorption of triacylglycerol in an olive oil loading test (in vivo test)
	In vitro pancreatic lipase activity inhibitory assay		Demonstrated inhibitory effect on pancreatic lipase activity
**Yuda et al., 2012 [99]**	In vitro pancreatic lipase inhibitory assay for black tea (*Camellia sinensis*) extracts rich in gallic acid	Theaflavin 3-*O*-gallate, theaflavin 3’-*O*-gallate, theaflavin 3,3’-*O*-gallate, epigallocatechin gallate, and epicatechin gallate	All extracts inhibited pancreatic lipase but extracts obtained at 100 to 140 °C showed the greatest lipase inhibition (IC50s of 0.9 to 1.3 μg/mL)
**Esposito et al., 2015 [11]**	High fat diet fed male C57BL/6J mice treated blackcurrant (*Ribes nigrum L*), rich in gallic acid, at 1% supplemented diet for 8 weeks	None	The extract reduced body weight gain and improved glucose metabolism
**Monika and Geetha, 2015 [124]**	High fat diet fed male Sprague Dawley rats treated with hydro-alcoholic fruit extract of avocado, rich in gallic acid, at 100 mg/kg body weight for 11 weeks	None	The extract reduced body mass index, adiposity index, total fat pad mass, blood cholesterol, triglycerides, and low-density lipoprotein. In addition, mRNA expression levels of fatty acid synthase, lipoprotein lipase, and leptin in adipose tissue was reduced
**Colantuono et al., 2016 [125]**	In vitro α-glucosidase, α-amylase and lipase inhibitory assays to assess pomegranate peels enriched cookies containing high levels of gallic acid and its derivatives	None	Showed inhibitory activity against α-glucosidase, α-amylase and α-lipase activities
**De Camargo et al., 2016 [126]**	In vitro antioxidant assays, as well as α-glucosidase and lipase inhibitory activities for phenolics from winemaking by-products rich in gallic acid	None	In addition to strong antioxidant potential, extracts showed inhibition of α-glucosidase and lipase activities
**Park et al., 2016 [127]**	High fat diet fed male C57BL/6 mice treated with an aqueous ethanol extraction of black tea, rich in gallic acid, at 100 and 300 mg/kg body weight for 8 weeks. 3T3-L1 adipocytes were exposed to 100 and 300 µg/mL during differentiation	None	Reduced body weight and body fat, improved fatty liver, regulated blood glucose, and decreased blood cholesterol. However, it did not have an effect on PPARγ protein expression
**Septembre-Malaterre et al., 2016 [128]**	3T3-L1 pre-adipocytes treated with pineapple and mango extracts, rich in garlic acid, at 25 µM for 1 h	None	Inhibited hydrogen peroxide induced production of reactive oxygen species
**Torabi and DiMarco, 2016 [129]**	3T3-F442A pre-adipocytes treated with grape powder extract, rich in gallic acid, at 125–500 mg GP/mL during differentiation period	None	The extract dose dependently induced adipocyte differentiation via upregulation of glucose transported (GLUT) 4, phosphatidylinositol-4,5- bisphosphate 3-kinase (PI3K) and adipogenic genes
**Pascual-Serrano et al., 2017 [130]**	High fat diet fed male Wistar rats treated with grape seed proanthocyanidin, rich in gallic acid, at 25 mg GSPE/kg body weight for 3 weeks	Gallic acid was used at 7 mg gallic acid/kg body weight for 3 weeks	Treatments did not reduce weight gain or reverse adiposity. However, the extract induced antihypertrophic and hyperplasic activities in white adipose tissue through enhancing perilipin-1 and fatty acid binding protein 4 expression and restoring adiponectin
**Simao et al., 2017 [131]**	In vitro α-amylase, α-glycosidase, lipase, and trypsin enzymes assays on aqueous extract from three cultivars of *Psidium guajava* L. (Pedro Sato, Paluma and Século XXI) rich in gallic acid	None	In presence of simulated gastric fluid, all cultivars showed increase in the inhibition of lipase and α-glycosidase, and decrease in inhibition of α-amylase and trypsin enzymes
**Ge et al., 2018 [132]**	The network-based pharmacological analysis was used to assess mulberry leaves rich in gallic acid	None	The extract regulated Tnf-α, PPARγ, glycogen synthase kinase-3 beta (GSK3B), insulin receptor substrate 1 (IRS1), interleukin 6 (IL-6) and other proteins involved in diabetes and obesity associated complications
**Sandoval-Gallegos et al., 2018 [133]**	High fat diet fed male Wistar rats treated with methanolic acid extract of *Mangifera indica* L. leaves, rich in gallic acid, at 100, 200 and 400 mg/kg for 32 days	None	In addition to increasing antioxidant capacity, the extract improved hyperlipidemic markers such as cholesterol, triglycerides, and atherogenic index
**Wu and Tian, 2018 [134]**	In vitro α-glucosidase, α-amylase and lipase inhibitory activity of flowers of pomegranate (*Punica granatum*) rich in gallic acid	Acarbose	The extract showed enhanced effect of suppress α-glucosidase, α-amylase, and lipase activities

**Table 4:** Overview of studies reporting on the ameliorative effects of other gallic acid-rich plants against obesity-associated complications.

Author, Year	Experimental Model, Dose Used, and Intervention Period	Comparative/Combination Therapy	Experimental Outcome and Proposed Mechanism
**Wu et al., 2010 [135]**	High fat diet fed male C57BL/6 mice treated with *Nelumbo nucifera* leaf extract-rich in gallic acid, supplemented at 0.5% in diet for 6 weeks	Simvastatin was used at 1 mg/kg body weight, while silymarin was used at 100 mg/kg body weight for 6 weeks	The extract performed comparable to simvastatin and silymarin in reducing body weight, body lipid accumulation, and activities of fatty acid synthase, glutamic oxaloacetic transaminase, and glutamic pyruvic transaminase
**Batista et al., 2014 [136]**	High fat diet fed male Sprague Dawley rats treated freeze-dried jaboticaba peel extract, rich in gallic acid, at 1%, 2% and 4% supplemented diet for 6 weeks	None	In addition to reducing circulating saturated free fatty acids, the extract prevented lipid peroxidation in the liver and increased its antioxidant defenses
**Foddai et al., 2014 [137]**	In vitro pancreatic triacylglycerol lipase, α-amylase and α-glucosidase inhibitory assays for *Limonium spp* (Plumbaginaceae) rich in epigallocatechins	Compared with acarbose, aqueous extracts of *L. contortirameum* and *L. virgatum*	All extract showed inhibitory activity on pancreatic triacylglycerol lipase, α-amylase and α-glucosidase
**Irondi et al., 2016 [138]**	In vitro pancreatic lipase and angiotensin 1-converting enzyme inhibitory assays for *Ocimum basilicum* extracts containing gallic acid	*Ocimum gratissimum* extracts	All extracts displayed high antioxidant properties. However, *Ocimum basilicum* displayed slightly lower activity than *Ocimum gratissimum* to inhibit pancreatic lipase and angiotensin 1-converting enzyme
**Abeysekera et al., 2017 [139]**	In vitro antilipidemic assays assessing potential of bark extracts of *Ceylon Cinnamon* rich in gallic acid	None	The extract showed inhibitory effect against HMG-CoA reductase, lipase, cholesterol esterase, and cholesterol micellization
**Donado-Pestana et al., 2018 [19]**	High fat diet fed male C57BL/6J mice treated with cagaita (*Eugenia dysenterica* DC.) extracts at 7 and 14 mg gallic acid equivalent (GAE)/kg body weight for 8 weeks	None	The extract protected against dyslipidemia, fasting hyperglycemia, and attenuated both hepatic gluconeogenesis and inflammation as observed by the expression of tumor necrosis factor alpha (TNF-α) and transcriptional factor NF-κB

**Table 5:** Human studies reporting on the therapeutic potential of gallic acid or gallic acid rich plants against obesity-associated complications.

Author, Year.	Experimental Model, Dose Used, And Intervention Period	Comparative/Combination Therapy	Experimental Outcome and Proposed Mechanism
**Roberts, 2006 [141]**	Obese human subjects receiving capsules containing 200 mg of gallic acid and 50 mg of a Chinese herbal decoction, three times a day for 24 weeks	None	Did not cause weight loss or a decrease in food intake in humans, principally due to the inability to achieve adequate serum levels
**Greenway et al., 2006 [142]**	Overweight women receiving number ten supplement (6 and mg/day), containing gallic acid, for 8 weeks	None	The supplement did not affect weight change; however had varied effect in food intake
**Heber et al., 2007 [143]**	Overweight human subjects received one or two pomegranate ellagitannin-enriched polyphenol extract capsules per day providing 710 mg (435 mg of gallic acid equivalents, GAEs) or 1420 mg (870 mg of GAEs) of extracts, respectively	None	Improved antioxidant activity through a significant reduction in thiobarbituric acid reactive substances
**Skrzypczak-Jankun and Jankun, 2010 [144]**	Plasma from human subjects treated with theaflavin digallate at 18 µM for 30 min	PAI-1 inhibitor PAI039 and epigallocatechin-3-gallate were used at 15 μM for 30 min	Inactivated plasminogen activator inhibitor type one (PAI-1)
**Kubota et al., 2011 [145]**	Pre-obese Japanese human subjects treated with water-soluble black Chinese (Pu-Erh) tea extract rich in gallic acid at 333 mg for 12 weeks	None	Exhibited significant effects in reducing the mean waist circumference, body mass index, and visceral fat values
**Hernández et al., 2015 [146]**	Obese patients undergoing biliopancreatic diversion received treatment with 2 courses of oral bismuth subgallate at 200 mg every 8 h for 12weeks, with a 4-week rest period	None	Improved the quality of life score of patients

## Abbreviations

AMPK	AMP-activated protein kinase
·OH	hydroxyl radical
O_2_^• −^	superoxide anion
C/EBP	CCAAT/enhancer-binding proteins
FAS	fatty acid synthase
FASL	FAS ligand
FFAs	free fatty acids
HFD	high fat diet
H_2_O_2_	hydrogen peroxide
iNOS	inducible nitric oxide synthase
IKK	inhibitor κB kinase
JNK	c-JUN NH2-terminal kinase
IRS-1	insulin receptor substrate 1
IL-6	interleukin
MCP-1	monocyte chemoattractant 1
NO	nitric oxide
NOX	nicotinamide adenine dinucleotide phosphate (NADPH) oxidase
NF-κB	nuclear factor kappa-light -chain-enhancer of activated B cells
PPAR	peroxisome proliferator-activated receptor
PGC1α	peroxisome proliferator activated receptor gamma coactivator 1 alpha
PI3K	phosphatidylinositol 3-kinase
PKC	phosphokinase C
Akt	protein kinase B
ROS	reactive oxygen species
SIRT1	NAD-dependent deacetylase sirtuin-1
T_H_17	T-helper cell 17
TNF-α	tumor necrosis factor alpha
p53	tumor protein 53
T2D	type 2 diabetes
